# Positive modulation of N-methyl-D-aspartate receptors in the mPFC reduces the spontaneous recovery of fear

**DOI:** 10.1038/s41380-022-01498-7

**Published:** 2022-04-14

**Authors:** Boyoung Lee, Santosh Pothula, Min Wu, Hyeyeon Kang, Matthew J. Girgenti, Marina R. Picciotto, Ralph J. DiLeone, Jane R. Taylor, Ronald S. Duman

**Affiliations:** 1 Department of Psychiatry, Yale School of Medicine, New Haven, CT USA; 2 Center for Cognition and Sociality, Institute for Basic Science, Daejeon, Republic of Korea

**Keywords:** Neuroscience, Diseases

## Abstract

N-methyl-D-aspartate receptor (NMDAR) modulators have recently received increased attention as potential therapeutics for posttraumatic stress disorder (PTSD). Here, we tested a novel NMDAR-positive modulator, NYX-783, in the following two rodent models of PTSD: an auditory fear-conditioning model and a single-prolonged stress (SPS) model. We examined the ability of NYX-783 to reduce subsequent fear-based behaviors by measuring enhanced fear extinction and reduced spontaneous recovery (spontaneous return of fear) in male mice. NYX-783 administration significantly reduced spontaneous recovery in both PTSD models and enhanced fear extinction in the SPS model. Furthermore, NYX-783 increased the NMDA-induced inward currents of excitatory and inhibitory neurons in the infralimbic medial prefrontal cortex (IL mPFC) and that the GluN2B subunit of NMDARs on pyramidal neurons in the IL mPFC is required for its effect on spontaneous recovery. The downstream expression of brain-derived neurotrophic factor was required for NYX-783 to achieve its behavioral effect. These results elucidate the cellular targets of NYX-783 and the molecular mechanisms underlying the inhibition of spontaneous recovery. These preclinical findings support the hypothesis that NYX-783 may have therapeutic potential for PTSD treatment and may be particularly useful for inhibiting spontaneous recovery.

## Introduction

Posttraumatic stress disorder (PTSD) is a neuropsychiatric disorder that occurs after experiencing or witnessing traumatic or life-threatening events [1]. In 2013, PTSD was revised and categorized as a trauma- and stressor-related disorder in the fifth edition of the Diagnostic and Statistical Manual of Mental Disorders (DSM-5) by the American Psychiatric Association. In the DSM-5, PTSD is defined by the following four symptoms: re-experiencing, avoidance, hyperarousal and negative alterations in mood and cognition [2]. Healthy individuals learn to respond to reminders of traumatic memories more adaptively. Failure to reduce fear leads to the development of PTSD psychopathology [3]. Selective serotonin reuptake inhibitors (SSRIs) are the only pharmacological class of antidepressant medications approved to treat PTSD. The limitations of SSRIs, such as delayed action and limited effectiveness in a small minority of patients [4], highlight an unmet need for more effective treatments for PTSD. Currently, cognitive behavioral therapy (CBT) has the greatest efficacy in treating PTSD symptoms; however, the nonresponse rates of PTSD patients to CBT are as high as 50% [5, 6]. In particular, therapies involving fear extinction learning, such as exposure therapy, cannot confer sustained, successful extinction, and the spontaneous recovery of fear occurs over time [7–10]. Therefore, spontaneous recovery after extinction has faced validity regarding clinical interventions for PTSD [11]. The synergy between antidepressant drugs and extinction training has been deemed necessary to persistently inhibit spontaneous recovery or fear renewal [12].

Glutamatergic transmission has been implicated in the pathophysiology of PTSD, particularly in the effects of N-methyl-D-aspartate receptor (NMDAR) signaling on the synaptic plasticity underlying learning and memory [13]. NMDARs comprise two GluN1 subunits and two GluN2 (A-D) or GluN3 (A, B) subunits. In adult forebrain regions, GluN2A and GluN2B are the main subunits forming receptor complexes with GluN1 at excitatory synapses. GluN2B-containing NMDARs play a preferential role in inducing synaptic plasticity, which is critical for the extinction of fear memories [14, 15]. Systemic injection of GluN2B-specific NMDAR antagonists ((RS)-3-(2-carboxypiperazin-4-yl)-propyl-1-phosphonic acid, ifenprodil) can impair the retention of fear extinction learning. GluN2B-containing NMDARs in both the amygdala and medial prefrontal cortex (mPFC) are also involved in reducing fear during extinction, whereas GluN2A-containing NMDARs play a greater role in the initial formation and/or stabilization of learned fear [15]. Rodent studies demonstrate that GluN2B subunit-containing NMDARs play pivotal roles in fear extinction learning.

The mPFC is critical for the acquisition and extinction of learned fear [16]. Activation of prelimbic (PL) regions of the mPFC is considered to drive fear learning, whereas activation of infralimbic (IL) regions of the mPFC inhibits learned fear [17]. Importantly, the intrinsic excitability of IL neurons increases during extinction consolidation and decreases during spontaneous recovery because of the return of learned fear over time, a situation that is similar to the behavior of IL neurons after fear conditioning [18]. Therefore, the IL region has been targeted to facilitate extinction and fear memory erasure. In particular, a previous study in which brain-derived neurotrophic factor (BDNF) or a BDNF-binding antibody (anti-BDNF) was infused into the IL showed that synaptic plasticity in this region of the mPFC is critical for fear memory extinction and spontaneous recovery [19].

Among rapid-acting antidepressant compounds, NMDAR modulators have recently received substantial attention for their potential in developing therapeutics for PTSD [20, 21]. In this study, we examined the effect of NYX-783, a novel NMDAR modulator, in a mouse model of PTSD. NYX-783 and NYX-2925 [22] were developed from a platform of spiro-β-lactam compounds that mimic some of the dipyrrolidine structural features of rapastinel (GLYX-13), and these are orally bioavailable compounds. These compounds, including NYX-783, are not canonical partial agonists or positive allosteric modulators of the glycine site of NMDAR because they facilitate NMDAR channel opening even in the absence of glycine [22]. These compounds act as glutamate coligands to positively modulate NMDAR activity, including all 4 NMDAR2A-D subtypes, with more preferential activation of NMDAR2B [22]. A phase II trial (ClinicalTrials.gov Identifier: NCT04044664) recently demonstrated the efficacy of NYX-783 in subjects with PTSD. Importantly, in preclinical studies, NYX-783 had no psychotomimetic side effects, offering an advantage over other rapid-acting antidepressant medications, such as ketamine. The goals of this study were to identify the cellular and molecular mechanisms underlying the behavioral effects of NYX-783 in preclinical models of PTSD, aiding the future development of more effective medications to treat this devastating disorder.

## Results

### NYX-783 reduces spontaneous recovery only when injected 1 h before the first extinction session in male and female mice

**Fig. 1:**
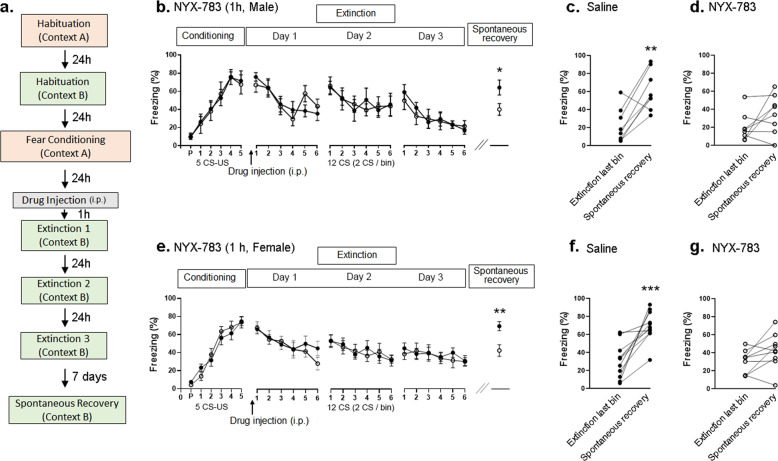
NYX-783 inhibits spontaneous recovery of learned fear in a conventional fear-conditioning model. **a** Schematic illustration of the fear-conditioning paradigm. The fear-conditioning protocol (5 CS (84 dB tone for 30 s) US (0.5 mA shock for 1 s) coupled protocol) was modified from a previous report from our laboratory [23]. A 10-min habituation to each chamber (fear conditioning and extinction) was included to ensure the formation and spontaneous recovery of fear memory [41, 42]. Mice were randomly assigned to two groups according to the freezing level during initial fear conditioning. After 24 h, the mice received an intraperitoneal (i.p.) injection of saline or NYX-783. One hour later, the first of three extinction sessions (12 tones with no shocks) was conducted in a context different from that of the original fear-conditioning session. The subsequent extinction sessions were conducted at 24 h intervals, with the three sessions spanning a total of three consecutive days. Seven days later, spontaneous recovery of fear was measured in the same extinction context to test the maintenance of extinction learning. NYX-783 (1 mg/kg; i.p.) treatment significantly inhibited spontaneous recovery after fear conditioning in both male and female mice. **b** Conditioning of male mice: freezing (% time) for each of the five CS–US pairing trials during fear conditioning in context A, demonstrating the establishment of CS–US pairing. P indicates the baseline freezing during 180 s of habituation in the chamber. Either saline or NYX-783 (1 mg/kg; i.p.) was injected 1 h before the first extinction trial. Day 1: freezing during the first day of extinction in context B. Twelve tones were delivered, and freezing from two tones was averaged (1 bin). Day 2: freezing during the second day of extinction in context B. Day 3: freezing during the third day of extinction in context B. Spontaneous recovery: freezing of a single CS presentation in context B performed 7 days after the last extinction. **c** Saline: spontaneous recovery. **d** NYX-783: Spontaneous recovery. *n* = 7–8 male mice per group. **e** Percentage of freezing in female mice after fear conditioning, extinction (day 1, day 2, day 3), and spontaneous recovery trials. **f** Saline: Spontaneous recovery. **g** NYX-783: spontaneous recovery. *n* = 9–11 female mice per group. **b**, **e** Conditioning and extinction: two-way ANOVA with Sidak’s multiple comparisons post hoc test. Spontaneous recovery: unpaired two-tailed *t* test (**c**, **d**, **f**, **g**) paired two-tailed *t* test *p < 0.05, **p < 0.01, ***p < 0.001, ****p < 0.0001. All the data are expressed as mean ± SEM. P preconditioned stimulus, CS conditioned stimulus, US unconditioned stimulus.

Failure to extinguish fearful and traumatic memories is a core symptom of PTSD. In addition, PTSD patients who have undergone successful exposure therapy remain susceptible to fear return after a period of time, a phenomenon known as “spontaneous recovery” [9]. Therefore, we tested the influence of NYX-783 on subsequent fear extinction and spontaneous recovery. The fear-conditioning protocol (Fig. 1a) used was modified from a previous report from our laboratory [23]. No significant differences in freezing were observed during the initial conditioning or across the extinction trials (Fig. 1b). However, compared with saline treatment, the administration of 1 mg/kg of NYX-783 1 h before the first extinction session significantly decreased freezing during the spontaneous recovery test (Fig. 1b), a finding that was not observed when NYX-783 (1 mg/kg) was injected 24 h before the first extinction session (Supplementary Fig. 1) or when ketamine (10 mg/kg) was injected 1 h before the first extinction session (Supplementary Fig. 2). Furthermore, the within-group analysis revealed the inhibition of fear return in the NYX-783-treated group (Fig. 1d and Supplementary Fig. 3a), whereas the saline group showed a significant increase in spontaneous recovery compared with the freezing level associated with the final bin during the extinction session on day 3 (Fig. 1c and Supplementary Fig. 3a).

We also tested a lower dose of NYX-783 (0.1 mg/kg) and found no differences in extinction learning or spontaneous recovery (Supplementary Fig. 4). In addition, we tested a weaker fear-conditioning protocol using three CS–US-coupled protocols to determine whether NYX-783 can facilitate extinction. A significant reduction in freezing was observed on day 2 of extinction (Supplementary Fig. 5), indicating that NYX-783 can facilitate fear extinction using this protocol. Although a trend was observed for reduced freezing during spontaneous recovery, the group difference in freezing during spontaneous recovery did not reach statistical significance, likely because of the lower freezing level during spontaneous recovery in the saline group. Because we were interested in determining whether NYX-783 could reduce spontaneous recovery, we continued to use the five CS–US protocols, as shown in Fig. 1.

Furthermore, we tested the efficacy of NYX-783 in female mice (Fig. 1e–g). Similar to the male group, no significant differences in freezing were observed during conditioning or extinction (Fig. 1e). The administration of NYX-783 (1 mg/kg or 0.1 mg/kg) 1 h before the first extinction session significantly decreased freezing in the spontaneous recovery session compared with that in the saline group (Fig. 1e and Supplementary Fig. 3b). A lower dose of NYX-783 (0.1 mg/kg) significantly facilitated extinction and reduced spontaneous recovery (Supplementary Fig. 6). Thus, both 1 mg/kg and 0.1 mg/kg of NYX-783 effectively reduced spontaneous recovery in female mice.

### NYX-783 promotes fear extinction and reduces spontaneous recovery of learned fear in a single-prolonged stress model in male mice

**Fig. 2:**
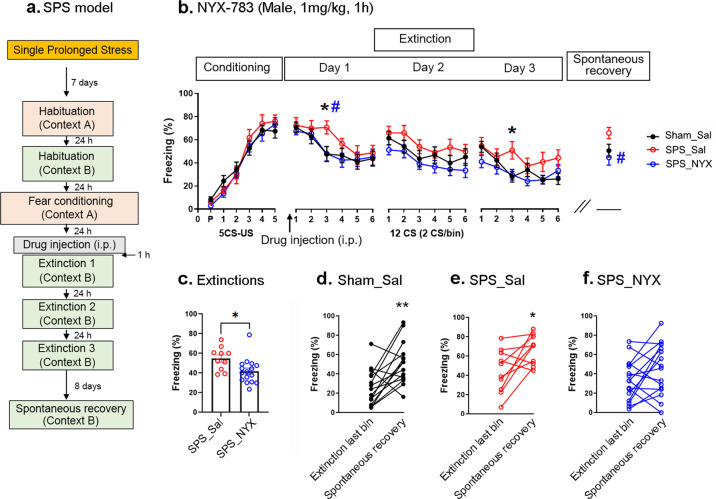
NYX-783 facilitates extinction and inhibits the spontaneous recovery of learned fear in an SPS mouse model of PTSD in male mice. **a** Schematic illustration of SPS and the fear-conditioning paradigm. NYX-783 (1 mg/kg; i.p.) treatment significantly reduced the % freezing in SPS-subjected mice in extinction trials and significantly inhibited spontaneous recovery compared with that in the SPS-vehicle (SPS_Sal) group. **b** Percentage of freezing in male mice after fear conditioning, extinction (day 1, day 2, day 3), and spontaneous recovery trials. **c** Average freezing percentages of extinctions. **d**–**f** Within-group comparisons of male mice. **d** Black closed circles indicate saline without SPS stress before fear conditioning (Sham_Sal). Red open circles indicate the saline group with SPS stress before fear conditioning (SPS_Sal). Blue open circles indicate the NYX-783-treated group with SPS stress before fear conditioning (SPS_NYX). *n* = 10–17 mice per group. **b** Conditioning and extinction: two-way ANOVA with Sidak’s multiple comparisons post hoc test. Spontaneous recovery: one-way ANOVA, Dunnett’s multiple comparisons post hoc test. *Comparisons between Sham_Sal and SPS_Sal, ^#^Comparisons between SPS_Sal and SPS_NYX. *^,#^*p* < 0.05 **c** unpaired two-tailed *t* test **d**–**f** paired two-tailed *t* test *p < 0.05, **p < 0.01. All the data are expressed as mean ± SEM. SPS single-prolonged stress, P preconditioned stimulus, CS conditioned stimulus, US unconditioned stimulus, Sham_Sal Sham_Saline, SPS_Sal SPS_Saline, SPS_NYX SPS_NYX-783.

To confirm the ability of NYX-783 to decrease the spontaneous recovery of learned fear, we employed another well-known rodent model of PTSD: the single-prolonged stress (SPS) model [24, 25]. One week after SPS, fear conditioning was performed (Fig. 2a), and the three groups were compared. Group 1 received saline and no SPS as a control (saline), Group 2 was subjected to SPS and injected with saline (SPS_Sal), and Group 3 was subjected to SPS and injected with 1 mg/kg of NYX-783 (SPS_NYX) 1 h before the first extinction session. Consistent with previous reports [24, 26], despite prior exposure to SPS, no significant difference in freezing was observed between the Sham_Sal and SPS_Sal groups or between the SPS_Sal and SPS_NYX groups during fear conditioning (Fig. 2b). Significant differences were found in extinction learning between the Sham_Sal and SPS_Sal groups and between the SPS_Sal and SPS_NYX groups. No differences were found in spontaneous recovery between the Sham_Sal and SPS_Sal groups, although a significant difference was observed in spontaneous recovery between the SPS_Sal and SPS_NYX groups (Fig. 2b). Analysis of the average freezing during the 3 days of the extinction sessions revealed a significant difference between the SPS_Sal and SPS_NYX groups (Fig. 2c), suggesting that NYX-783 administration facilitated extinction. Within-group analysis revealed that the Sham_Sal and SPS_Sal groups showed a significant increase in spontaneous recovery (Fig. 2d, e), but the SPS_NYX group showed no significant spontaneous recovery (Fig. 2f). Taken together, these results suggest that the administration of NYX-783 significantly inhibited spontaneous recovery even when the animals were exposed to stress before fear conditioning.

### *Grin2b* knockdown in pyramidal neurons in the mPFC blocks the ability of NYX-783 to inhibit the spontaneous recovery of learned fear in male mice

**Fig. 3:**
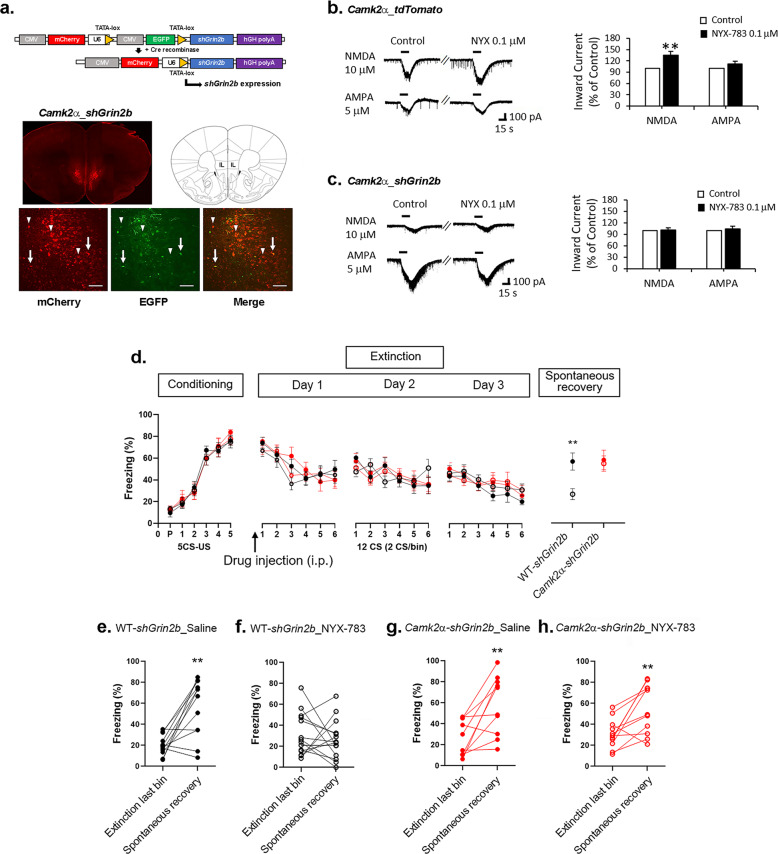
*Grin2b* knockdown in glutamatergic neurons blocks the effect of NYX-783 on the inhibition of the spontaneous recovery of learned fear in male mice. **a** Top: schematic of the Cre-dependent *Grin2b* knockdown (*AAV2shGrin2b*) construct before and after Cre recombination to generate the active construct. Bottom: representative images of Cre-dependent *AAV2shGrin2b* virus expression in the IL mPFC in *Camk2α-Cre* transgenic mice (*Camk2α-Cre_AAV2shGrin2b*). Arrows indicate cells expressing both mCherry and EGFP. Arrowheads indicate Cre-recombined cells expressing only mCherry. **b** *Camk2α*_*tdTomato* (*Camk2α-Cre::Ai14*): representative traces of NMDA- and AMPA-induced inward currents before and after 0.1 µM NYX-783. NMDA-induced inward current (*n* = 16 cells, 8 mice) and AMPA-induced inward current (*n* = 8 cells, 7 mice). Bar graph (% of control): Application of 0.1 µM NYX-783 significantly increased NMDA (10 µM)-induced inward currents but did not increase AMPA (5 µM)-induced inward currents of pyramidal neurons in the IL mPFC. **c** *Camk2α*_*shGrin2b* (*Camk2α-Cre_ AAV2shGrin2b*): representative traces of NMDA- and AMPA-induced inward currents before and after 0.1 µM NYX-783 treatment. NMDA-induced inward current (*n* = 6 cells, 4 mice) and AMPA-induced inward current (*n* = 7 cells, 4 mice). Application of 0.1 µM NYX-783 did not increase the NMDA-induced inward currents of *Camk2α*-positive neurons expressing Cre-dependent *AAV2shGrin2b* in the IL mPFC. AMPA-induced inward currents were not changed. Bar graph: NYX-783 (1 mg/kg; i.p.) treatment significantly inhibited spontaneous recovery only in WT mice infused with Cre-dependent *AAV2shGrin2b* virus, whereas this effect of NYX-783 was blocked in *Camk2α-Cre* mice after *Grin2b* knockdown. Bar = 100 µm. **d** Percentage of freezing in male mice after fear conditioning, extinction (day 1, day 2, day 3), and spontaneous recovery trials. **e**–**h** Within-group comparisons of male mice. **e** Black closed circles indicate saline injection with Cre-dependent *AAV2shGrin2b* virus expression in wild-type littermates (WT-*shGrin2b*_Saline). **f** Black open circles indicate NYX-783 injection with Cre-dependent AAV2shGrin2b virus expression in wild-type littermates (WT-*shGrin2b*_NYX-783). **g** Red closed circles indicate saline injection with Cre-dependent *AAV2shGrin2b* virus expression in *Camk2α-Cre* mice (*Camk2α-shGrin2b*_Saline). **h** Red open circles indicate NYX-783 injection with Cre-dependent *AAV2shGrin2b* virus expression in *Camk2α-Cre* mice (*Camk2α-shGrin2b*_NYX-783). **d** Conditioning and extinction: three-way ANOVA. Spontaneous recovery: two-way ANOVA with Tukey’s multiple comparisons post hoc test. **e**–**h** Paired two-tailed *t* test **p < 0.01. All the data are expressed as mean ± SEM. P preconditioned stimulus, CS conditioned stimulus, US unconditioned stimulus.

Among the GluN2 subunits, GluN2B, which is encoded by *Grin2b*, has been suggested to be involved in extinction [15]. Therefore, we focused on manipulating *Grin2b* to test the efficacy of NYX-783 in spontaneous recovery in different cell types using a viral vector-mediated Cre-dependent knockdown system. First, the virus was injected into WT or *Camk2α-Cre* transgenic mice. Strong mCherry expression was observed in the IL mPFC after the infusion of Cre-dependent *AAV2shGrin2b* (Fig. 3a). Most mCherry-expressing cells did not express eGFP, validating Cre-mediated recombination and indicating that Cre-dependent *AAV2shGrin2b* was expressed in pyramidal neurons (Fig. 3a, high-magnification image). Some cells, likely GABAergic interneurons, expressed both mCherry and eGFP (Fig. 3a, high-magnification image).

First, to validate the ability of NYX-783 to act as an NMDAR-positive modulator, we measured NMDAR-mediated currents in mPFC slices. To perform cell type-specific electrophysiological recordings, we used brain slices from *Camk2α_tdTomato* double transgenic mice (*Camk2α-Cre::Ai14*) in which excitatory neurons expressed the red fluorescent protein tdTomato. The application of NMDA (10 μM) increased the NMDAR-mediated inward currents, which were significantly enhanced in the presence of NYX-783 (0.1 μM) compared with saline (Fig. 3b). However, the α-amino-3-hydroxy-5-methyl-4-isoxazolepropionic acid (AMPA) receptor-mediated inward currents were not altered following NYX-783 treatment (Fig. 3b), suggesting that the effects of NYX-783 were specific to NMDARs. The knockdown of *Grin2b* in pyramidal neurons completely abolished the increased NMDA-inward current induced by NYX-783 (Fig. 3c), suggesting that GluN2B was an essential subunit mediating the NMDAR currents induced by NYX-783.

Three weeks after surgery, fear conditioning was performed in the following four groups: Group 1: WT mice + saline (WT-*shGrin2b*_saline); Group 2: WT mice + NYX-783 (WT-*shGrin2b_*NYX-783); Group 3: *Camk2α-Cre* + saline (*Camk2α-shGrin2b_*saline); and Group 4: *Camk2α-Cre* + NYX-783 (*Camk2α-shGrin2b_*NYX-783). No significant differences in fear acquisition or extinction (Fig. 3d) were observed among the four groups. However, *Grin2b* knockdown in *Camk2α-Cre* mice completely blocked the effect of NYX-783 on spontaneous recovery (Fig. 3d–h). Both the between-group and within-group analyses (Supplementary Fig. 3d) showed that the ability of NYX-783 to prevent fear return was abolished by *Grin2b* knockdown in *Camk2α−*expressing neurons.

### *Grin2b* knockdown in GABAergic interneurons in the mPFC does not alter the effect of NYX-783 on the spontaneous recovery of learned fear in male mice

**Fig. 4:**
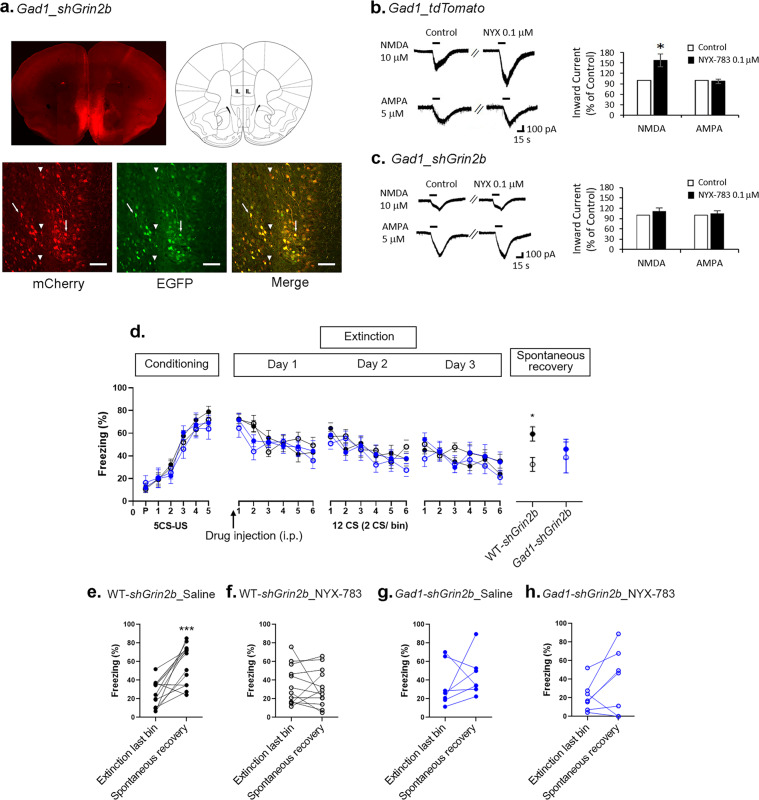
*Grin2b* knockdown in GABAergic interneurons does not block the effect of NYX-783 on the inhibition of the spontaneous recovery of learned fear in male mice. **a** Representative images of Cre-dependent *AAV2shGrin2b* virus expression in IL mPFC in *Gad1-Cre* mice (*Gad1-Cre_AAV2shGrin2b*). Arrows indicate cells expressing both mCherry and EGFP. Arrowheads indicate Cre-recombined cells expressing only mCherry. **b** Representative traces of NMDA- and AMPA-induced inward currents before and after 0.1 µM NYX-783 in GABAergic interneurons. NMDA-induced inward current (*n* = 10 cells, 6 mice) and AMPA-induced inward current (*n* = 9 cells, 6 mice). Inward current (% of control) is shown as bars. Application of 0.1 µM NYX-783 significantly increased NMDA (10 µM)-induced inward currents but did not increase AMPA (5 µM)-induced inward currents in major GABAergic interneurons (*Gad_tdTomato*) in the IL mPFC. **c** Representative traces of NMDA- and AMPA-induced inward currents before and after 0.1 µM NYX-783 treatment. NMDA-induced inward current (*n* = 8 cells, 6 mice) and AMPA-induced inward current (*n* = 6 cells, 4 mice). Application of 0.1 µM NYX-783 did not increase the NMDA-induced inward current of GABAergic interneurons (*Gad1*-positive neurons) expressing Cre-dependent *AAV2shGrin2b* in the IL mPFC. AMPA-induced inward currents were not changed. **b**, **c** Two-tailed *t* test *p < 0.05. All the data are expressed as mean ± SEM. Bar = 100 µm. **d** Percentage of freezing in male mice after fear conditioning, extinction (day 1, day 2, day 3), and spontaneous recovery trials. **e**–**h** Within-group comparisons of male mice. **e** Black closed circles indicate saline injection with Cre-dependent *AAV2shGrin2b* virus expression in wild-type littermates (WT-*shGrin2b*_Saline). **f** Black open circles indicate NYX-783 injection with Cre-dependent *AAV2shGrin2b* virus expression in wild-type littermates (WT-*shGrin2b*_NYX-783). **g** Blue closed circles indicate saline injection with Cre-dependent *AAV2shGrin2b* virus expression in *Gad1-Cre* mice (*Gad1-shGrin2b*_Saline). **h** Blue open circles indicate NYX-783 injection with Cre-dependent *AAV2shGrin2b* virus expression in *Gad1-Cre* mice (*Gad1-shGrin2b*_NYX-783). *n* = 7–14 mice per group. **d** Conditioning and extinctions: three-way ANOVA. Spontaneous recovery: two-way ANOVA with Tukey’s multiple comparisons post hoc test. **e**–**h** Paired two-tailed *t* test *p < 0.05, ***p < 0.001. All the data are expressed as mean ± SEM. P preconditioned stimulus, CS conditioned stimulus, US unconditioned stimulus.

Next, we tested the role of GluN2B in GABAergic interneurons in conditioned fear, extinction and spontaneous recovery by injecting Cre-dependent *AAV2shGrin2b* into the IL mPFC of *Gad1-Cre* mice. Consistent with the observation that pyramidal cells constitute ~70% of mPFC neurons [27], most cells in the virally infused *Gad1-Cre* mouse line expressed both mCherry and eGFP. However, a substantial number of cells expressed only mCherry, suggesting that efficient recombination occurred in *Gad1*-positive GABAergic interneurons in the mPFC (Fig. 4a). Similar to *Camk2a_tdTomato*, compared with saline, NYX-783 (0.1 μM) enhanced NMDA (10 μM)-mediated currents in Gad1-positive neurons (Fig. 4b), whereas no change in AMPA currents was observed following NYX-783 treatment (Fig. 4b), suggesting that NYX-783 could also increase NMDA currents in GABAergic interneurons in the IL mPFC. NYX-783 no longer enhanced NMDA-inward currents in Gad1-positive neurons in which *Grin2b* was knocked down (Fig. 4c). These data show that the GluN2B subunit is an essential subunit mediating the NMDAR currents induced by NYX-783 in both glutamatergic neurons and GABAergic interneurons.

Three weeks after surgery, the following four groups of mice were evaluated in the fear-conditioning paradigm: WT-*shGrin2b_*Saline, WT-*shGrin2b_*NYX-783, *Gad1-shGrin2b_*Saline and *Gad1-shGrin2b_*NYX-783 groups. No differences in fear learning or extinctions were observed among any of the groups. The WT-*shGrin2b_*Saline group significantly differed from the WT-*shGrin2b_*NYX-783 group in spontaneous recovery. No difference was observed between the WT-*shGrin2b_*NYX-783 and *Gad1-shGrin2b*_NYX-783 groups (Fig. 4d). In the within-group analyses, among the groups, only the WT-*shGrin2b_*Saline group showed a significant fear return (Fig. 4e and Supplementary Fig. 3e). The saline-treated *Gad1-AAV2shGrin2b* group (*Gad1-shGrin2b_*Saline) did not show fear return (Fig. 4g), indicating that *Grin2b* knockdown in GABAergic interneurons alone can reduce spontaneous recovery. Altogether, NYX-783 reduced spontaneous recovery of learned fear in WT mice (Fig. 4f) and the *Gad1-AAV2shGrin2b* group (Fig. 4h).

### BDNF in the infralimbic (IL) mPFC after extinction is required for the effect of NYX-783 on the spontaneous recovery of learned fear in male mice

**Fig. 5:**
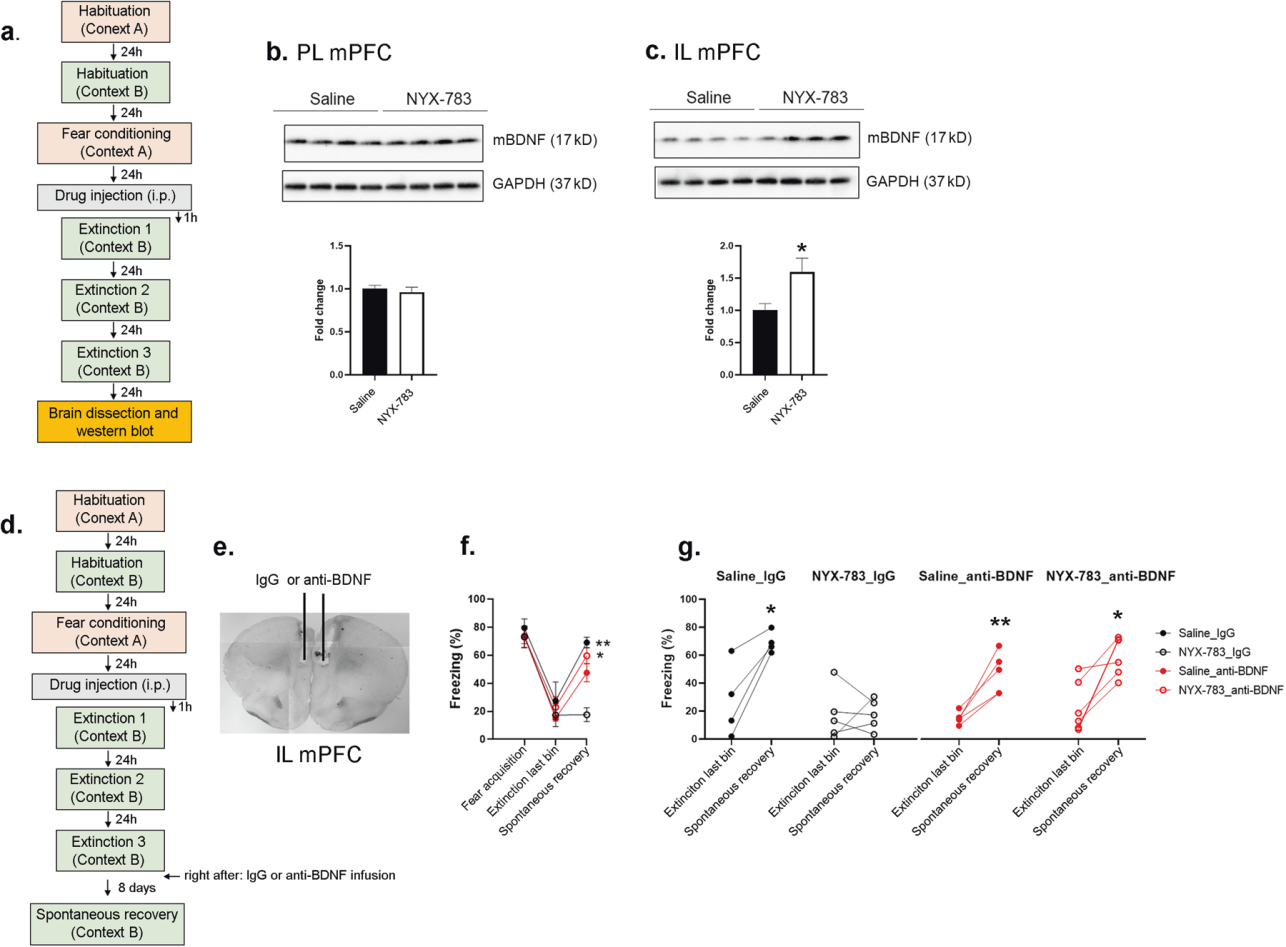
Function-blocking BDNF antibody (anti-BDNF) blocks the effect of NYX-783 on the inhibition of spontaneous recovery of learned fear in male mice. **a** Schematic illustration of the fear-conditioning paradigm and sample collection. **b** Representative western blot images and bar graph of BDNF in the PL mPFC. **c** Representative western blot images and bar graph of BDNF in the IL mPFC. *n* = 4–6 mice per group. All the data are expressed as mean ± SEM. *p < 0.05. **d** Schematic illustration of the fear-conditioning paradigm. Infusion of anti-BDNF nAb immediately after the extinction trial (on day 3) prevented the effect of NYX-783 on the inhibition of spontaneous fear return. **e** Representative image of cannulation. **f** Fear acquisition in male mice: Freezing (% time) for the last CS from the five CS–US pairing trials during fear conditioning in context A. Either saline or NYX-783 (1 mg/kg; i.p.) was injected 1 h before the first extinction. Extinction last bin: average freezing of the last two tones during the extinction on day 3. Spontaneous recovery: % freezing of single CS presentation in context B performed 8 days after the last extinction. **g** Graphs of within-group comparisons in male mice. Black closed circles indicate Saline injection with control IgG infusion (Saline_IgG). Black open circles indicate NYX-783 injection with control IgG infusion (NYX-783_IgG). Red closed circles indicate saline injection with anti-BDNF mPFC infusion (saline_anti-BDNF). Red open circles indicate NYX-783 injection with anti-BDNF infusion (NYX-783_anti-BDNF). *n* = 4–6 mice per group. **b**, **c** Unpaired two-tailed *t* test. **f** Three-way ANOVA with Sidak’s multiple comparisons post hoc test. **g** Paired two-tailed *t* test *p < 0.05, **p < 0.01. All the data are expressed as mean ± SEM.

Several studies have shown the association between BDNF expression in extinction learning and spontaneous recovery [28–30]. Therefore, we evaluated the effect of NYX-783 on BDNF expression after extinction training (Fig. 5 and Supplementary Fig. 7). We determined the protein levels in the PL and IL mPFC separately. No differences were found in the BDNF levels in the PL between the saline- and NYX-783-treated groups (Fig. 5b). However, we found significant differences in the BDNF expression levels in the IL between the saline- and NYX-783-treated groups 2 h (Supplementary Fig. 7) and 24 h after the final extinction trial (Fig. 5c and Supplementary Fig. 7). We also compared the BDNF expression level with that in the no shock control group mice, which were placed in fear-conditioning chambers and extinction chambers for the same amount of time without CS or US. Only the NYX-783-treated groups exhibited significantly increased BDNF expression in the IL mPFC (Supplementary Fig. 7).

Next, we tested the contribution of BDNF expression to the effects of NYX-783 on the reduced spontaneous recovery of learned fear. Based on a previous study [31], we infused anti-BDNF ~5 min after extinction to evaluate the requirement of BDNF in the IL mPFC to inhibit spontaneous recovery (Fig. 5d, e). Four groups were compared: saline IgG, NYX-783_IgG, saline anti-BDNF and NYX-783_anti-BDNF. All four groups were equally distributed with respect to freezing following fear acquisition and during the last bin of the last extinction session (Fig. 5f). Between-group analysis revealed a significant difference between the Saline_IgG and NYX-783_IgG groups during spontaneous recovery, confirming the effectiveness of NYX-783 in reducing spontaneous recovery with control IgG infusion in the IL mPFC. No significant difference was found between the saline_IgG and saline_anti-BDNF groups. However, freezing during spontaneous recovery in the NYX-783_anti-BDNF group was significantly higher than that in the NYX-783_IgG group (Fig. 5f), suggesting that BDNF inactivation by anti-BDNF infusion into the IL mPFC abolished the effect of NYX-783 on spontaneous recovery. The within-group analysis showed that among the groups, only the NYX-783_IgG group exhibited inhibited spontaneous recovery (Fig. 5g).

## Discussion

In this study, NYX-783, a novel NMDAR-positive modulator, reduced fear return during a test of spontaneous recovery both in the conventional auditory fear-conditioning paradigm and following single-prolonged stress in male mice. *Grin2b* knockdown in either excitatory or inhibitory neurons prevented the effect of NYX-783 on NMDA-inward currents in mPFC slices. *Grin2b* knockdown in pyramidal neurons in the IL mPFC blocked the effect of NYX-783 on the spontaneous recovery of learned fear, and BDNF expression was required for the ability of NYX-783 to reduce the spontaneous recovery of fear.

NYX-783 significantly reduced spontaneous recovery of fear in both male and female mice in the conventional auditory fear-conditioning model. NYX-783 was more effective in females, suggesting differential sensitivity of NMDAR modulators to males and females. In line with this, previous studies have reported that female rodents were more sensitive to NMDAR antagonist (MK-801)-induced excitotoxic damage [32], and ketamine treatment mediated physiological and behavioral responses [33, 34], suggesting increased NMDA receptor sensitivity, which could be due to higher expression of NMDA receptors in female rodents [35]. However, further studies are needed to address the mechanisms underlying the increased sensitivity of female rodents to NYX-783.

NYX-783 also reduced spontaneous recovery in the SPS mouse model of PTSD, suggesting that this compound can inhibit the spontaneous recovery of learned fear even after exposure to an additional significant stressor. Interestingly, we observed significant facilitation of fear extinction following NYX-783 administration in the SPS model. SPS impairs extinction learning and extinction memory retention, primarily through dysregulation of the HPA axis but also via attenuation of glutamate levels and NMDAR activation in the IL mPFC [25]. These data suggest that NYX-783 likely rescues the reduction in fear extinction induced by SPS via the activation of NMDARs in the IL. Although we observed a significant decrease in spontaneous recovery with NYX-783, NYX-783 may delay spontaneous recovery. Because we focused on spontaneous recovery 7 or 8 days after the last extinction, further studies may need to test spontaneous recovery at later time points to confirm the effect of NYX-783 on spontaneous recovery at different time points.

Although some conflicting results have been reported regarding whether ketamine’s behavioral effects in stress-related paradigms are mediated through glutamatergic neurons or GABAergic interneurons in the mPFC, recent studies suggest that ketamine acts through GluN2B-containing NMDARs on GABAergic interneurons but not glutamatergic neurons [34–36]. Thus, we tested whether NYX-783 also exerts an effect via a similar mechanism by knocking down *Grin2b* in excitatory and inhibitory neurons in the IL mPFC. Supplementary Fig. 8 summarizes the molecular mechanisms underlying the actions of NYX-783 on inhibiting spontaneous recovery, particularly by activating GluN2B-containing NMDARs. We suggest that the activation of GluN2B-containing NMDARs on postsynaptic glutamatergic neurons by NYX-783 (Supplementary Fig. 8a) may be crucial for its effect on fear return during spontaneous recovery because *Grin2b* knockdown in glutamatergic neurons blocked the effect of NYX-783 on the reduction in the spontaneous recovery of fear (Supplementary Fig. 8b). Interestingly, saline injection in *Gad1-shGrin2b* mice also blocked fear return during spontaneous recovery, indicating that *Grin2b* knockdown in GABAergic interneurons alone facilitates extinction consolidation potentially by disinhibition-induced pyramidal neuron activation and increased glutamate release from presynaptic glutamatergic neurons (Supplementary Fig. 8c). We observed no inhibitory effect of NYX-783 on spontaneous recovery when *Grin2b* was knocked down in GABAergic neurons. The reason is likely that the knockdown of *Grin2b* in interneurons occluded the effect of NYX-783, suggesting that NYX-783 may act via GluN2B in interneurons. However, whether NYX-783 acts via GluN2B in interneurons to reduce spontaneous recovery remains unclear because *Grin2b* knockdown in interneurons without NYX-783 already shows low freezing during spontaneous recovery. Because of this floor effect, we may not see a further reduction in freezing with NYX-783 during spontaneous recovery even if NYX-783 acts via GluN2B on glutamatergic neurons. The role of NYX-783 in GABAergic neurons must be explored further. Since we found that NYX-783 enhances NMDA currents in both glutamatergic neurons and GABAergic neurons, which neuronal type is primarily responsible for the effect of NYX-783 is unclear. An offsetting effect may exist such that NYX-783 preferentially acts on GluN2B in glutamatergic neurons. Alternatively, because glutamatergic neurons are the major output or projection neurons to amygdala inhibitory neurons to modulate extinction, NYX-783-mediated GluN2B activation in glutamatergic neurons is more critical for behavioral output, a possibility that must be tested in the future.

Based on these findings, we propose that GluN2B may serve as the initial cellular trigger activating the downstream signaling cascades that are reinforced during extinction to enhance synaptic plasticity in the IL mPFC and strengthen extinction consolidation. To confirm this hypothesis, we examined the synaptic plasticity marker BDNF. Interestingly, BDNF was significantly increased only in the IL but not in the PL mPFC. The involvement of the IL mPFC, but not the PL mPFC, in extinction consolidation has been demonstrated in other studies [37]. Such changes may be essential to maintain extinction memory and, thus, reduce the return of fear behavior observed during the subsequent spontaneous recovery session.

In summary, this study suggests a possible action of NYX-783 through activating GluN2B-containing NMDARs and BDNF expression, maintaining extinction memories and leading to reduced spontaneous recovery of learned fear. Here, we also demonstrate that *Grin2b* knockdown in GABAergic interneurons reduces spontaneous fear recovery potentially by disinhibiting pyramidal neurons, similar to ketamine’s action. Together, these findings suggest that NYX-783, a novel NMDAR-positive modulator, may be an effective medication for PTSD. Although clinical studies of this compound are ongoing, these findings suggest that the development of NMDAR modulators may be a viable strategy to treat PTSD.

## Methods

### Animals

For all behavioral experiments except the viral infusion studies, male and female C57BL/6J mice (Jackson Laboratories, Bar Harbor, ME) were used. For the viral studies, male and female transgenic mice and WT (C57BL/6J background) littermates were obtained from in-house breeders. *Gad1-Cre* mice were originally obtained from Marina Picciotto (Yale School of Medicine) [38], and *Camk2α-Cre* mice were obtained from Günter Schütz (German Cancer Research Center, Heidelberg, Germany) [39]. For the electrophysiology experiments to evaluate the effects of NYX-783 on NMDA- or AMPA-induced inward currents, *Camk2α-tdTomato*, *Gad1-tdTomato*, and Cre-dependent *AAV2shGrin2b* virus-infused *Camk2α-Cre* and *Gad1-Cre* male and female mice were used. All the studies were performed with adult mice (10–16 weeks of age). The animals were housed in a standard ventilated rack under a 12-h light/12-h dark cycle with ad libitum access to water and rodent chow. All the experiments were performed during the 12-h light cycle. Animal use and procedures adhered to NIH guidelines and were approved by the Yale University Animal Care and Use Committees and Institutional Animal Care and Use Committees of IBS (Daejeon, Korea).

### Pharmacological agents

All the compounds were diluted in 0.9% saline. Ketamine (10 mg/kg; Sigma–Aldrich, St. Louis, MO) or NYX-783 (0.1 mg/kg or 1 mg/kg; Aptinyx, Evanston, IL) was intraperitoneally injected 24 h after fear conditioning, and 0.9% saline was injected as a vehicle control.

### Fear conditioning

Auditory fear conditioning was performed as previously described [23], with minor modifications. Mice were randomly assigned to each group according to the freezing level during initial fear conditioning. In addition, freezing was measured using an automated computer analysis system (Video Freeze, SOF-843).

### Single-prolonged stress

SPS was conducted as previously described [24]. After SPS, the mice were left undisturbed in their home cages for 7 days.

### shRNA and surgical procedures

For the knockdown of *Grin2b* in specific neuronal cell types, we used a validated Cre-dependent adeno-associated virus *AAV2shGrin2b* construct as reported by Gerhard et al. [34]. Cre-dependent *AAV2shGrin2b* virus (0.3 μl) was infused at 0.1 μl/min into the IL mPFC using the following coordinates: AP, 1.9 mm; ML, 0.4 mm; DV, −3.1 mm. For the knockdown experiment, behavioral experiments and analyses were conducted without randomization. For behaviors, investigators were blind to the types of virus injected.

### Brain slice electrophysiology

Electrophysiology was performed as described previously [34].

### Infusion of BDNF neutralizing antibody (anti-BDNF nAb)

Infusion of anti-BDNF nAb was performed immediately after the last extinction training (within 5–10 min). An infusion cannula (28 gauge; Plastics One, Roanoke, VA) was cut to extend 0.2 mm beyond the implanted guide cannula targeting the IL mPFC (AP, 1.9 mm; ML, 0.4 mm; DV, −2.8 mm). The cannula was left in place for 5 min before removal to allow diffusion of the drug. IgG (0.3 μl/min) or anti-BDNF nAb (0.3 μg/side; 1 μg/μl; Chemicon, Temecula, CA) was bilaterally infused as described previously [40].

### Western blot analysis

Western blot analysis was performed as previously described [1] using BDNF (1:2,000; GeneTex, Irvine, CA, USA) and GAPDH (1:5000; Cell Signaling, Danvers, MA, USA) antibodies. Signal detection was performed using the LAS-2000 system (LAS-2000; Fuji, Tokyo, Japan) with enhanced chemiluminescence (Western-CDP star; PerkinElmer Life Sciences, Norwalk, CT, USA). Densitometric analysis of immunoreactivity for each protein was conducted using NIH ImageJ software.

### Statistical analysis

All the results were included unless determined to be statistical outliers (>2 SD from the mean). The data were analyzed using GraphPad Prism 9 (GraphPad Software Inc., California). Comparisons between two groups were determined by Student’s *t* test To analyze freezing in the fear conditioning and extinction trials, two-way ANOVA with Sidak’s multiple comparisons post hoc test was performed. For within-group comparisons, one-way ANOVA with Tukey’s multiple comparisons post hoc test was performed. For comparisons among four groups (see Figs. 3 and 4), three-way ANOVA with Tukey’s multiple comparisons post hoc test was performed. For three group comparisons during spontaneous recovery in Fig. 2, one-way ANOVA with Dunnett’s multiple comparisons post hoc test was performed. All statistical analyses were performed using GraphPad Prism 9.0. We performed a statistical post hoc power analysis using the G*Power 3 calculator. Although the effective sample size could not be achieved for the two-tailed *t* test (effect size = 0.50; *α* = 0.05; 1–*β*  =  0.80; total sample size = 128) and ANOVA (effect size=0.50; *α* = 0.05; 1–*β* = 0.80; total sample size = 48), significant differences were observed in several behavioral tests. All the values indicated are mean ± SEM, and statistical significance is represented as asterisks at *p* values <0.05 (*), <0.01 (**), <0.001 (***), and <0.0001 (****).

## Supplementary information

Supplementary information

Supplementary Table 1.

Supplementary Figure 1.

Supplementary Figure 2.

Supplementary Figure 3.

Supplementary Figure 4.

Supplementary Figure 5.

Supplementary Figure 6.

Supplementary Figure 7.

Supplementary Figure 8.
