# Supplementary material for: Positive modulation of N-methyl-D-aspartate receptors in the mPFC reduces the spontaneous recovery of fear
Source: Mol Psychiatry. 2022 Apr 14;27(5):2580–9. doi: 10.1038/s41380-022-01498-7 (PMC9135632; doi:10.1038/s41380-022-01498-7)
Supplement: Supplementary file 1 — Supplementary information [file 41380_2022_1498_MOESM1_ESM.docx]

***Supplemental Information***

**Positive modulation of N-methyl-D-aspartate receptors in the mPFC reduces the spontaneous recovery of fear**

**Supplemental Material and Methods**

### **Fear conditioning**

Auditory fear conditioning was performed as previously described (1), with minor modifications. Briefly, 2 days before fear conditioning, mice were habituated in context A, the conditioning context, for 10 min and habituated to the room for 2 h. On 1 day before fear conditioning, mice were habituated in context B, the extinction chamber, for 10 min and habituated to the room for 2 h. On the fear conditioning day, mice were placed in the conditioning context (context A), and after a 180 s acclimation period, they received five pairings of the CS and US. The CS tone (84 dB, 2 kHz, 5 ms rise/fall time) was presented for 30 s and co-terminated with a brief US foot shock (1 s, 0.5 mA). The inter-tone interval (interval from tone onset to the next tone onset) ranged from 60 to 180 s. The conditioning chambers were cleaned between subjects with 70% ethanol. The time spent freezing during delivery of the CS tone was scored and presented as a percentage. Subjects were evenly distributed according to freezing level from conditioning and were matched on CS tone freezing levels. Then, the mice were assigned to the saline, ketamine or NYX-783 groups. The next day, each mouse received a single injection of ketamine, NYX-783 or 0.9 w/v saline. One hour or twenty-four hours after drug treatment, the ketamine, NYX-783 and vehicle-treated groups were placed in context B and extinction learning or training sessions began. Following a 3 min acclimation period, mice received 12 non-reinforced presentations of the CS (30 s, 84 dB, inter-tone interval: 60–90 s) in the novel context. Between trials, the chambers were cleaned with 70% ethanol. This procedure was repeated over the next 2 days to produce 3 separate days of extinction training sessions, and 7 or 8 days later, spontaneous recovery was assessed in a single session by returning the subjects to context B (Med Associates). Freezing, which is expressed as a percentage of time spent freezing during the 30-s tone period (unless otherwise noted), was measured using an automated computer analysis system (Video Freeze, SOF-843).

**Single prolonged stress**

Animals were randomly assigned into three groups (Sham_Sal, SPS_Sal, and SPS_NYX), and sham mice were left in their cages without handling. SPS was conducted as previously described (2). Briefly, the mice were restrained for 2 h in 50 mL polypropylene conical tubes with a nose hole for ventilation. After immobilization, each mouse was placed in a clear acrylic cylinder filled with water (24°C ± 1°C, 18 cm depth) and forced to swim for 20 min. Following 15 min of recuperation, the mice were exposed to diethyl ether until loss of consciousness and then left undisturbed in their home cages for 7 days.

**Brain slice electrophysiology**

Electrophysiology was performed as described previously (3). Briefly, brain slices containing the mPFC were prepared from male and female mice (8 to 16 weeks old). Brains were placed in artificial cerebrospinal fluid (ACSF) (pH 7.35–7.38) equilibrated with 95% O2/5% CO2, and coronal slices of 300-μm thickness containing the mPFC were transferred to the fixed stage of an Olympus BX50WI scope (Tokyo, Japan) for whole-cell recording. The chamber was continuously perfused with normal ACSF at a rate of 2 to 3 ml/min, and its temperature was maintained at 33 ± 0.5°C. Patch pipettes (3–5 MΩ) were pulled from glass tubing with a Flaming-Brown Horizontal Puller. The pipette solution contained the following: 115 mM K gluconate, 5 mM KCl, 2 mM MgCl2, 2 mM Mg-ATP, 2 mM Na2 ATP, 10 mM Na2 -phosphocreatine, 0.4 mM Na2 GTP, and 10 mM HEPES (pH 7.33). The neurons were visualized by video microscopy under a microscope (40x infrared (IR) lens) with IR differential interference contrast (IR/DIC). Whole-cell recordings were performed with an Axoclamp-2B amplifier. NMDA (10 μM)- and AMPA (5 μM)-induced inward currents were tested with no added magnesium in the ACSF.

**References**

1. Girgenti MJ, Ghosal S, LoPresto D, Taylor JR, and Duman RS. Ketamine accelerates fear extinction via mTORC1 signaling. Neurobiol Dis. 2017;100:1-8.

2. Yamamoto S, Morinobu S, Fuchikami M, Kurata A, Kozuru T, and Yamawaki S. Effects of single prolonged stress and D-cycloserine on contextual fear extinction and hippocampal NMDA receptor expression in a rat model of PTSD. Neuropsychopharmacology. 2008;33(9):2108-2116.

3. Gerhard DM, Pothula S, Liu RJ, Wu M, Li XY, Girgenti MJ, et al. GABA interneurons are the cellular trigger for ketamine's rapid antidepressant actions. J Clin Invest. 2020;130(3):1336-49.

**Supplemental Figure Legends**

**Supplementary Fig. 1. NYX-783 does not facilitate extinction and spontaneous recovery when injected 24 h before the first extinction session in male mice.** No significant changes in percentage freezing during extinction and spontaneous recovery trials were observed after NYX-783 (1 mg/kg, i.p.) administration 24 h prior to extinction trial (Day 1). (a) Schematic illustration of the fear conditioning paradigm. (b) Percentage of freezing in male mice after fear conditioning, extinction (Day 1, Day 2, Day 3), and spontaneous recovery trials. (c) Bar graph showing within-group comparisons of male mice. n=8-11 mice per group. (b) Conditioning and extinction: 2-way ANOVA with Sidak’s multiple comparisons post hoc test. Spontaneous recovery: unpaired two-tailed t test. (c) one-way ANOVA, Tukey’s multiple comparisons post hoc test. *, p<0.05, **, p<0.01. All data are the mean ± SEM. Abbreviations: P, pre-conditioned stimulus; CS, conditioned stimulus; US, unconditioned stimulus

**Supplementary Fig. 2. Ketamine does not facilitate extinction and spontaneous recovery in male mice when injected 1 h before the first extinction session.** No **s**ignificant changes in percentage freezing during the extinction and spontaneous recovery trials were observed after ketamine (10 mg/kg, i.p.) treatment 1 h prior to extinction trial (Day 1). (a) Percentage of freezing in male mice after fear conditioning, extinction (Day 1, Day 2, Day 3), and spontaneous recovery trials. (b) Bar graph showing within-group comparisons of male mice. n=7-9 mice per group. (a) Conditioning and extinction: 2-way ANOVA with Sidak’s multiple comparisons post hoc test. Spontaneous recovery: unpaired two-tailed t test. (b) one-way ANOVA, Tukey’s multiple comparisons post hoc test. *, p<0.05, **, p<0.01, ***, p<0.001. All data are the mean ± SEM. Abbreviations: P, pre-conditioned stimulus; CS, conditioned stimulus; US, unconditioned stimulus

**Supplementary Fig. 3. Bar graphs.** (a) Fig. 1b, Male, 1 mg/kg, 1 h (injected 1 h before the first extinction) (b) Fig. 1e, Female, 1 mg/kg, 1 h (c) Fig. 2b, SPS model, Male, 1 mg/kg, 1 h (d) Fig. 3d, *Camk2α-shGrin2b*, Male, 1 mg/kg, 1 h (e) Fig. 4d, *Gad1-shGrin2b*, 1 mg/kg, 1 h (f) Fig. 5f, anti-BDNF in the IL mPFC, Male, 1 mg/kg, 1 h. (a, b, c, d, e) one-way ANOVA, Tukey’s multiple comparisons post hoc test. (f) Conditioning and extinction: 2-way ANOVA with Sidak’s multiple comparisons post hoc test. All data are the mean ± SEM.*, p<0.05, **, p<0.01, ***, p<0.001, ****, p<0.0001. All data are the mean ± SEM. Abbreviations: P, pre-conditioned stimulus; CS, conditioned stimulus; US, unconditioned stimulus.

**Supplementary Fig. 4. Lower dose of NYX-783 does not facilitate extinction and spontaneous recovery in male mice.** No **s**ignificant changes in percentage freezing were observed during extinction and spontaneous recovery trials after NYX-783 (0.1 mg/kg, i.p.) administration 1 h prior to extinction trial (Day 1). (a) Percentage of freezing in male mice after fear conditioning, extinction (Day 1, Day 2, Day 3), and spontaneous recovery trials. (b) Bar graph showing within-group comparisons of male mice. n=7-9 mice per group. (a) Conditioning and extinction: 2-way ANOVA with Sidak’s multiple comparisons post hoc test. Spontaneous recovery: unpaired two-tailed t test. (b) one-way ANOVA, Tukey’s multiple comparisons post hoc test. *, p<0.05, **, p<0.01. All data are the mean ± SEM. Abbreviations: P, pre-conditioned stimulus; CS, conditioned stimulus; US, unconditioned stimulus

**Supplementary Fig. 5. NYX-783 facilitates extinction but does not reduce spontaneous recovery in male mice with a 3 CS-US protocol.** (a) Percentage of freezing in male mice after the fear conditioning, extinction (Day 1, Day 2, and Day 3), and spontaneous recovery trials. (b) Within-group comparisons of male mice receiving a saline injection. n=18 mice. (c) Within-group comparisons of male mice receiving NYX-783 injection. n=18 mice. (a) Conditioning and extinction: 2-way ANOVA with Sidak’s multiple comparisons post hoc test. Spontaneous recovery: unpaired two-tailed t test. (b, c) paired two-tailed t test. *, p<0.05. All data are the mean ± SEM.

**Supplementary Fig. 6. NYX-783 facilitates extinction and significantly reduces spontaneous recovery in female mice.** A significant reduction in percentage freezing was observed on Day 3 of extinction, and a significant reduction in percentage freezing was observed in the spontaneous recovery trial after the treatment with NYX-783 (0.1 mg/kg; i.p.) 1 h prior to the extinction trial (Day 1). (a) Percentage freezing in female mice after fear conditioning, extinction (Day 1, Day 2, Day 3), and spontaneous recovery trials. (b) Bar graph showing within-group comparisons of female mice. n=9-11 mice per group. (a) Conditioning and Extinctions: 2-way ANOVA with Sidak’s multiple comparisons post hoc test. Spontaneous recovery: unpaired two-tailed t test. (b) one-way ANOVA, Tukey’s multiple comparisons post hoc test. *, p<0.05, **, p<0.01, ****, p<0.0001. All data are the mean ± SEM. Abbreviations: P, pre-conditioned stimulus; CS, conditioned stimulus; US, unconditioned stimulus.

**Supplementary Fig. 7. NYX-783 significantly increases BDNF expression in the IL mPFC 2 hours and 24 hours after the final extinction session.** (a) Western blot image (b) Bar graph. n=4-6 mice per group. (b) one-way ANOVA, Tukey’s multiple comparisons post hoc test. *, p<0.05, ***, p<0.001. All data are the mean ± SEM.

**Supplementary Fig. 8. Summary of the molecular mechanisms underlying the actions of NYX-783 on the inhibition of spontaneous recovery of learned fear.** (a) NYX-783 activates GluN2B-containing NMDARs on glutamatergic neurons and likely triggers persistent increases in BDNF levels in the IL mPFC that result in the inhibition of spontaneous recovery after fear conditioning. (b) Cell-type specific *Grin2b* knockdown in excitatory neurons in the IL mPFC blocks the effect of NYX-783 on spontaneous recovery. (c) *Grin2b* knockdown in inhibitory neurons likely triggers disinhibition-mediated glutamate release and facilitates the indirect activation of pyramidal neurons in the IL mPFC, resulting in a baseline reduction in spontaneous recovery.
