# Supplementary material for: Positive modulation of N-methyl-D-aspartate receptors in the mPFC reduces the spontaneous recovery of fear
Source: Mol Psychiatry. 2022 Apr 14;27(5):2580–9. doi: 10.1038/s41380-022-01498-7 (PMC9135632; doi:10.1038/s41380-022-01498-7)
Supplement: Supplementary file 2 — Supplementary Table 1. [file 41380_2022_1498_MOESM2_ESM.docx]

**Supplementary table 1. Summary statistics**

| **Figure** | **Samples** | **Name of test** | **Statistics** |
| --- | --- | --- | --- |
| Figure 1b | Male, Conditioning | 2-way anova | F (1, 12)=0.003024, p=0.9570 |
| Figure 1b | Male, Day 1 | 2-way anova | F (1, 12)=0.0796, p=0.7826 |
| Figure 1b | Male, Day 2 | 2-way anova | F (1, 12)=0.0003278, p=0.9859 |
| Figure 1b | Male, Day 3 | 2-way anova | F (1, 12)=0.1015, p=0.7555 |
| Figure 1b | Male, Spontaneous Recovery | t test | t=2.287, df=12, p=0.0411 |
| Figure 1c | Male, Saline | paired t test | q=9.972, df=6, p<0.0001 |
| Figure 1d | Male, NYX-783 | paired t test | q=2.111, df=6, p=0. 0792 |
| Figure 1e | Female, Conditioning | 2-way anova | F(1, 18)=0.06220, p=0.8059 |
| Figure 1e | Female, Day 1 | 2-way anova | F (1, 18)=0.3297, p=0.5729 |
| Figure 1e | Female, Day 2 | 2-way anova | F (1, 18)=0.001801, p=0.9666 |
| Figure 1e | Female, Day 3 | 2-way anova | F (1, 18)=0.1030, p=0.7520 |
| Figure 1e | Female, Spontaneous Recovery | t test | t=3.330, df=18, p=0.0037 |
| Figure 1f | Female, Saline | paired t test | q=5.025, df=10, p=0.0005 |
| Figure 1g | Female, NYX-783 | paired t test | q=2.179, df=8, p=0.0610 |
| Figure 2b | Male, Conditioning  (Sham_Sal and SPS_Sal ) | 2-way anova | F(1, 23)=0.07063, p=0.7928 |
| Figure 2b | Male, Conditioning  (SPS_Sal and SPS_NYX) | 2-way anova | F(1, 23)=0.07063, p=0.7928 |
| Figure 2b | Male, Day 1  (Sham_Sal and SPS_Sal) | 2-way anova | bin3: t=2.642, df=22.91, p=0.0290 |
| Figure 2b | Male, Day 1  (SPS_Sal and SPS_NYX) | 2-way anova | bin3: t=2.699, df=23.47, p=0.0252 |
| Figure 2b | Male, Day 3  (Sham_Sal and SPS_Sal) | 2-way anova | bin3: t=2.592, df=15.59, p=0.0395 |
| Figure 2b | Male, Spontaneous Recovery (Sham_Sal and SPS_Sal) | one-way anova | t=1.662, df=38, p=0.1749 |
| Figure 2b | Male, Spontaneous Recovery (SPS_Sal and SPS_NYX) | one-way anova | t=2.331, df=38, p=0.0447 |
| Figure 2c | Extinctions | t test | t=2.566, df=24, p=0.0170 |
| Figure 2d | Sham_Sal | paired t test | q=3.606, df=14, p=0.0029 |
| Figure 2e | SPS_Sal | paired t test | q=2.759, df=9, p=0.0222 |
| Figure 2f | SPS_NYX | paired t test | q=1.700, df=15, p=0.1098 |
| Figure 3b | Camk2a_tdTomato_NMDA | t test | t=3.565, df=15, p=0.0028 |
| Figure 3b | Camk2a_tdTomato_AMPA | t test | t=1.539, df=7, p=0.1677 |
| Figure 3c | Camk2a_shGrin2b_NMDA | t test | t=0.2161, df=5, p=0.8375 |
| Figure 3c | Camk2a_shGrin2b_AMPA | t test | t=0.6151, df=6, p=0.5611 |
| Figure 3d | Conditioning | 3-way anova | F (1, 43)=0.01782, p=0.8944 |
| Figure 3d | Day1 | 3-way anova | F (1, 43)=0.04416, p=0.8346 |
| Figure 3d | Day2 | 3-way anova | F (1, 43)=0.2954, p=0.5896 |
| Figure 3d | Day3 | 3-way anova | F (1, 43)=0.6837, p=0.4129 |
| Figure 3d | Spontaneous recovery (WT-shGrin2b) | 2-way anova | t=3.127, df=43, p=0.0063 |
| Figure 3d | Spontaneous recovery (Camk2a-shGrin2b) | 2-way anova | t=0.3149, df=43, p=0.9397 |
| Figure 3e | WT-shGrin2b_Saline | paired t test | q=12.44, df=11, p<0.0001 |
| Figure 3f | WT-shGrin2b_NYX-783 | paired t test | q=6.138, df=13, p=0.0059 |
| Figure 3g | Camk2a-shGrin2b_Saline | paired t test | q=8.908, df=9, p=0.0010 |
| Figure 3h | Camk2a-shGrin2b_NYX-783 | paired t test | q=9.117, df=10, p=0.0005 |
| Figure 4b | Gad1_tdTomato | t test | t=3.064, df=9, p=0.0135 |
| Figure 4c | Gad1_shGrin2b | t test | t=0.451, df=8, p=0.6640 |
| Figure 4d | Conditioning | 3-way anova | F (1, 36) = 0.05589, p=0.8145 |
| Figure 4d | Day1 | 3-way anova | F (1, 36) = 0.3605, p=0.5520 |
| Figure 4d | Day2 | 3-way anova | F (1, 36) = 0.2040, p=0.6542 |
| Figure 4d | Day3 | 3-way anova | F (1, 36) = 1.581, p=0.2167 |
| Figure 4d | Spontaneous recovery (WT-shGrin2b) | 2-way anova | t=2.666, df=35, p=0.0229 |
| Figure 4d | Spontaneous recovery (Gad1-shGrin2b) | 2-way anova | t=0.5437, df=35, p=0.8320 |
| Figure 4e | WT-shGrin2b_Saline | paired t test | q=5.155, df=13, p=0.0002 |
| Figure 4f | WT-shGrin2b_NYX-783 | paired t test | q=0.5724, df=11, p=0.5786 |
| Figure 4g | Gad1-shGrin2b_Saline | paired t test | q=0.8302, df=6, p=0.4382 |
| Figure 4h | Gad1-shGrin2b_NYX-783 | paired t test | q=1.570, df=6, p=0.1675 |
| Figure 5b | PL mPFC | t test | t=0.5354, df=6, p=0.6116 |
| Figure 5c | IL mPFC | t test | t=2.498, df=6, p=0.0467 |
| Figure 5f | Saline_IgG vs Saline_anti-BDNF | 3-way anova | q=21.62, df=7.588, p=0.3312 |
| Figure 5f | Saline_IgG vs NYX-783_IgG | 3-way anova | q=11.76, df=6.936, p=0.0017 |
| Figure 5f | NYX-783_anti-BDNF vs NYX-783_IgG | 3-way anova | q=7.956, df=8.986, p=0.0087 |
| Figure 5g | Saline_IgG | paired t test | q=4.124, df=3, p=0.0259 |
| Figure 5g | NYX-783_IgG | paired t test | q=0.03519, df=4, p=0.9736 |
| Figure 5g | Saline_anti-BDNF | paired t test | q=5.211, df=4, p=0.0065 |
| Figure 5g | NYX-783_anti-BDNF | paired t test | q=3.903, df=5, p=0.0111 |
