# Supplementary figures and images for: Positive modulation of N-methyl-D-aspartate receptors in the mPFC reduces the spontaneous recovery of fear
Source: Mol Psychiatry. 2022 Apr 14;27(5):2580–9. doi: 10.1038/s41380-022-01498-7 (PMC9135632; doi:10.1038/s41380-022-01498-7)

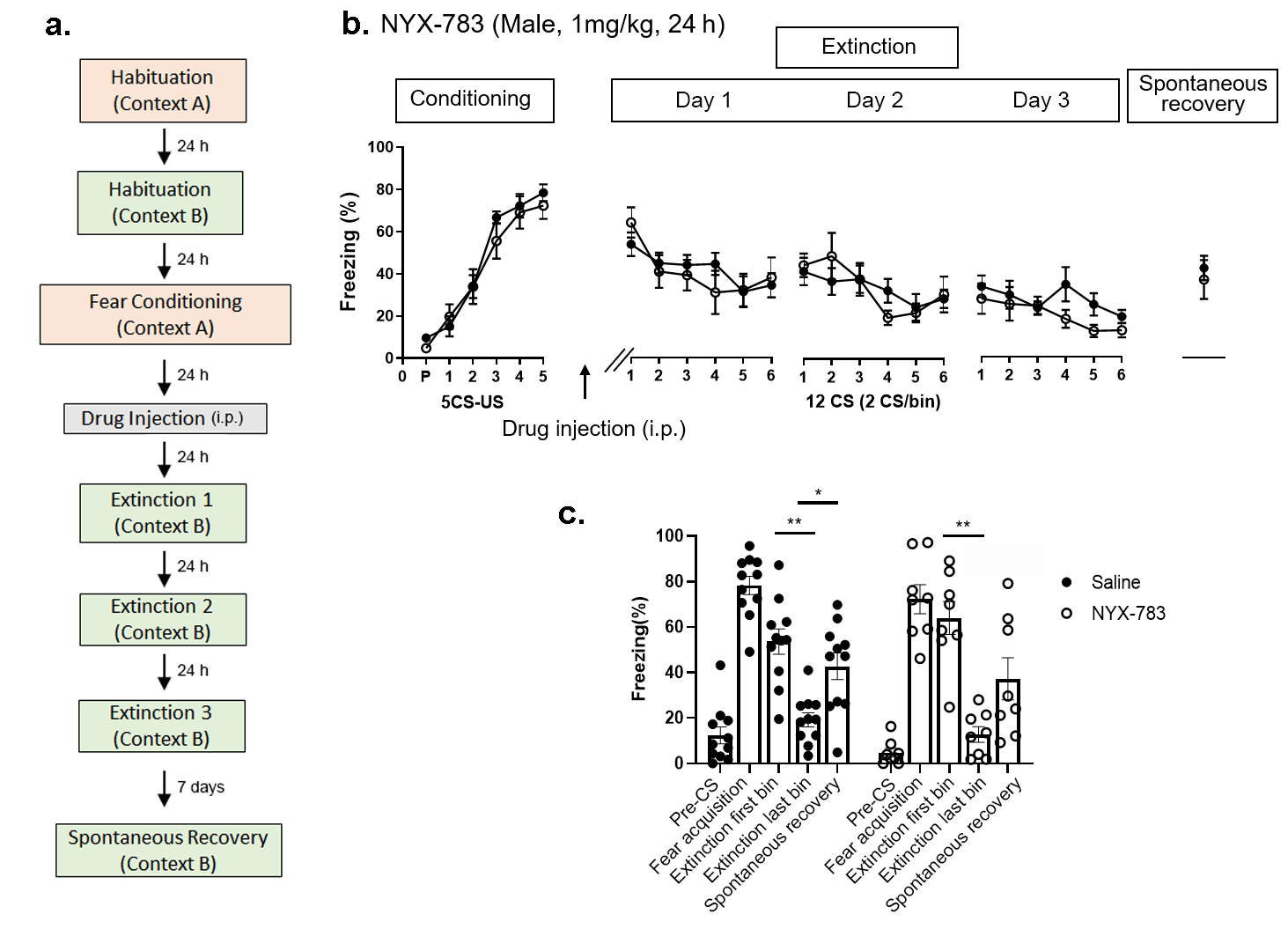

Supplement: Supplementary file 3 — Supplementary Figure 1. [file 41380_2022_1498_MOESM3_ESM.tif]

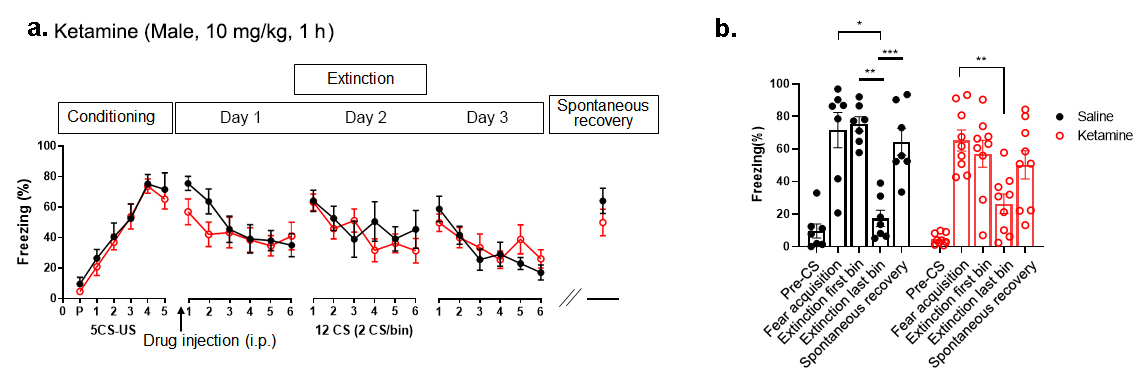

Supplement: Supplementary file 4 — Supplementary Figure 2. [file 41380_2022_1498_MOESM4_ESM.tif]

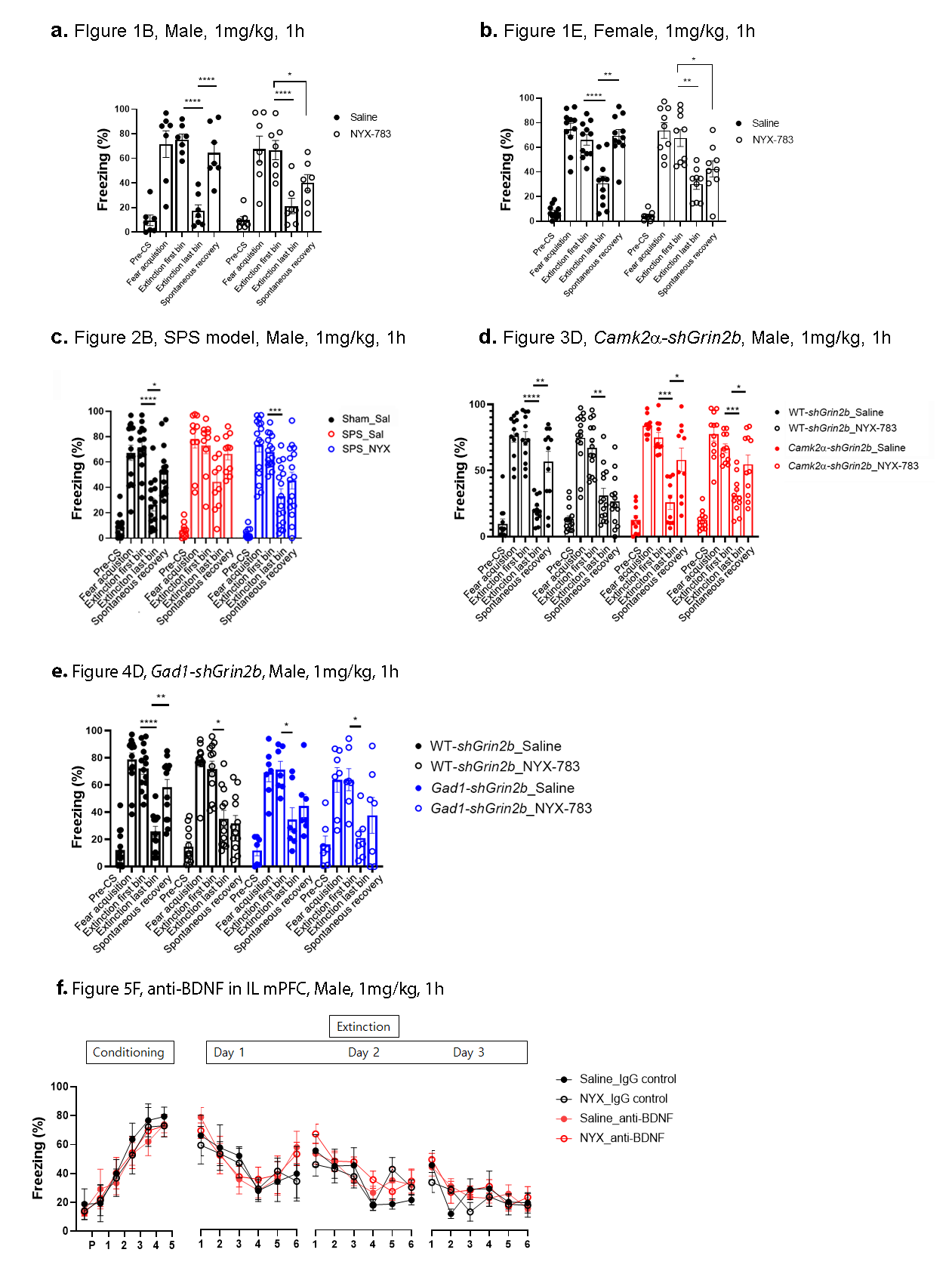

Supplement: Supplementary file 5 — Supplementary Figure 3. [file 41380_2022_1498_MOESM5_ESM.tif]

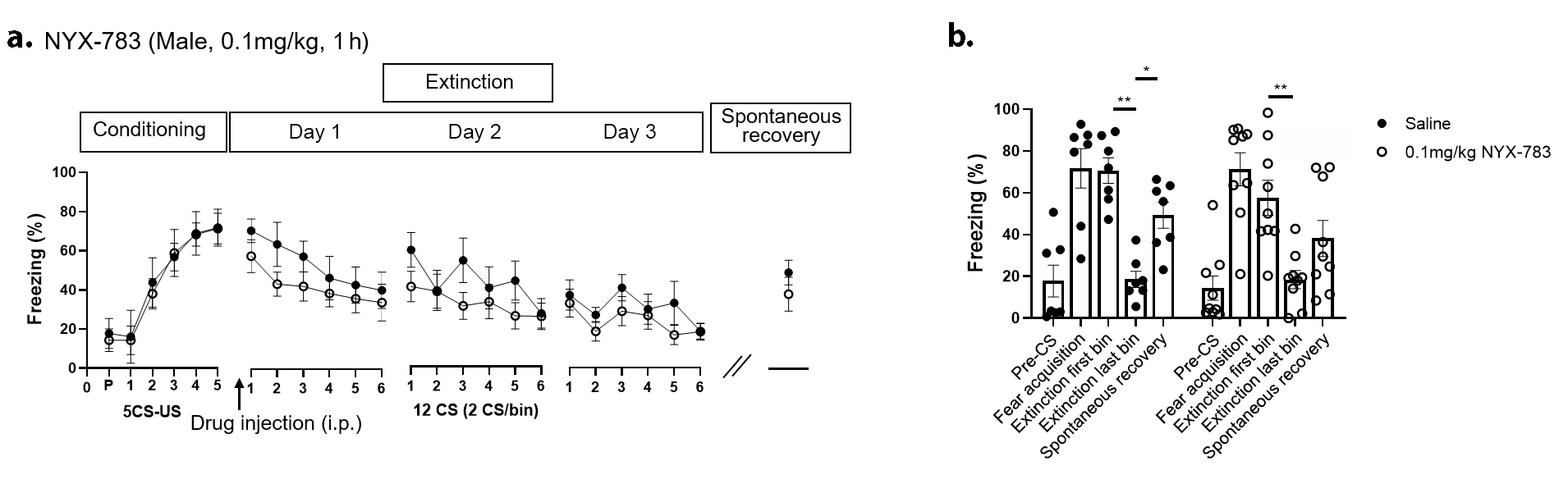

Supplement: Supplementary file 6 — Supplementary Figure 4. [file 41380_2022_1498_MOESM6_ESM.tif]

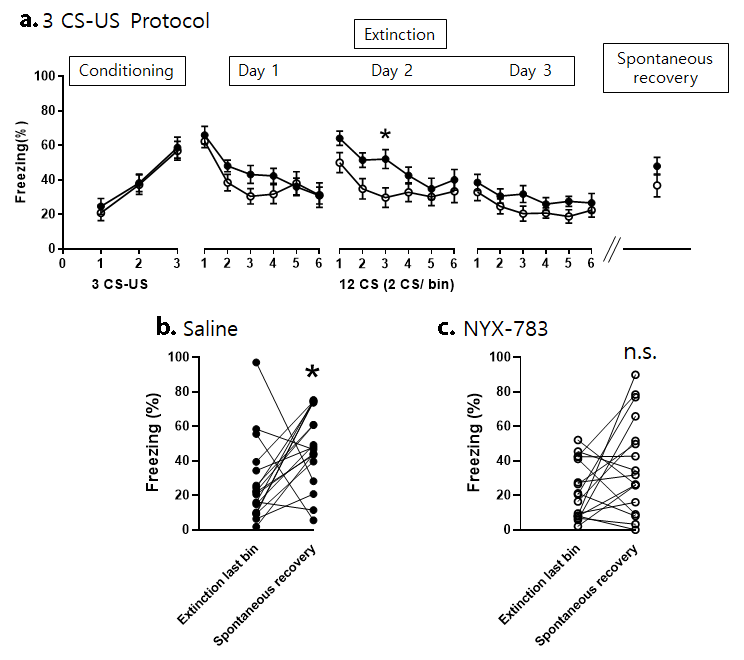

Supplement: Supplementary file 7 — Supplementary Figure 5. [file 41380_2022_1498_MOESM7_ESM.tif]

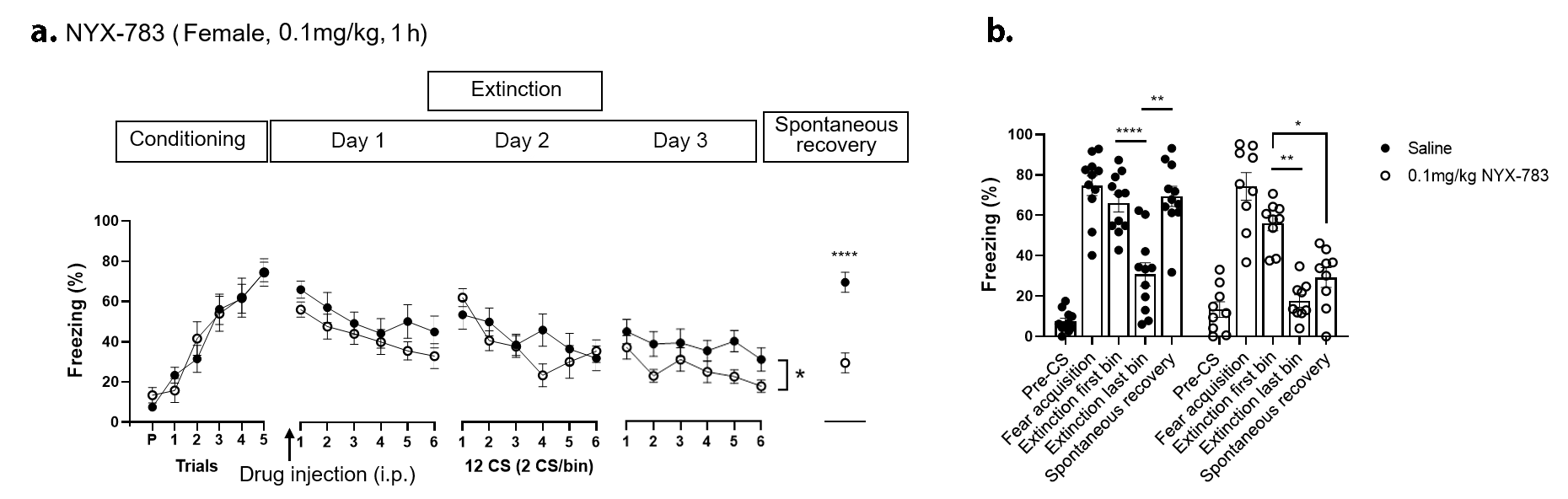

Supplement: Supplementary file 8 — Supplementary Figure 6. [file 41380_2022_1498_MOESM8_ESM.tif]

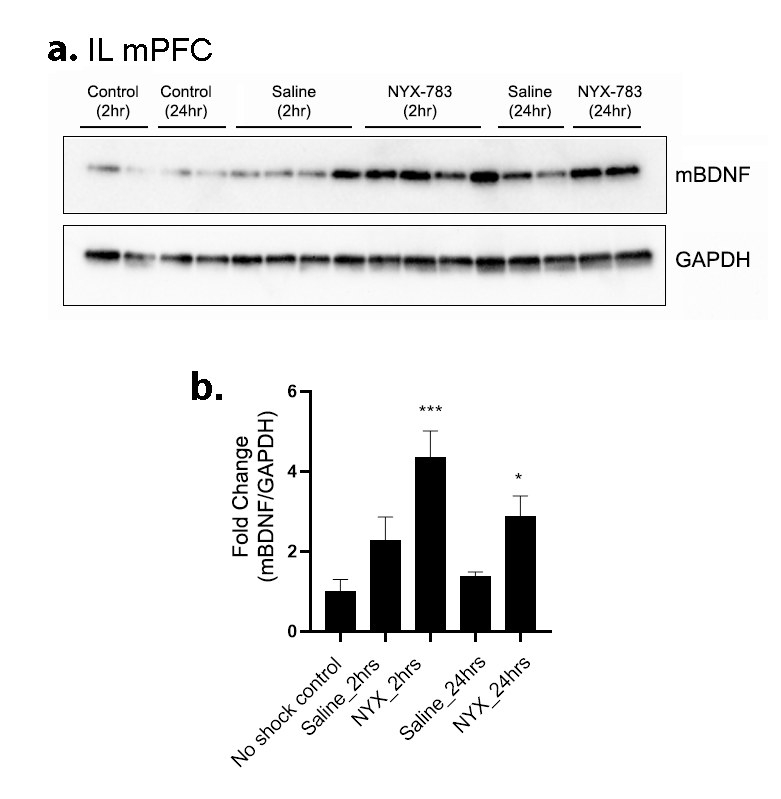

Supplement: Supplementary file 9 — Supplementary Figure 7. [file 41380_2022_1498_MOESM9_ESM.tif]

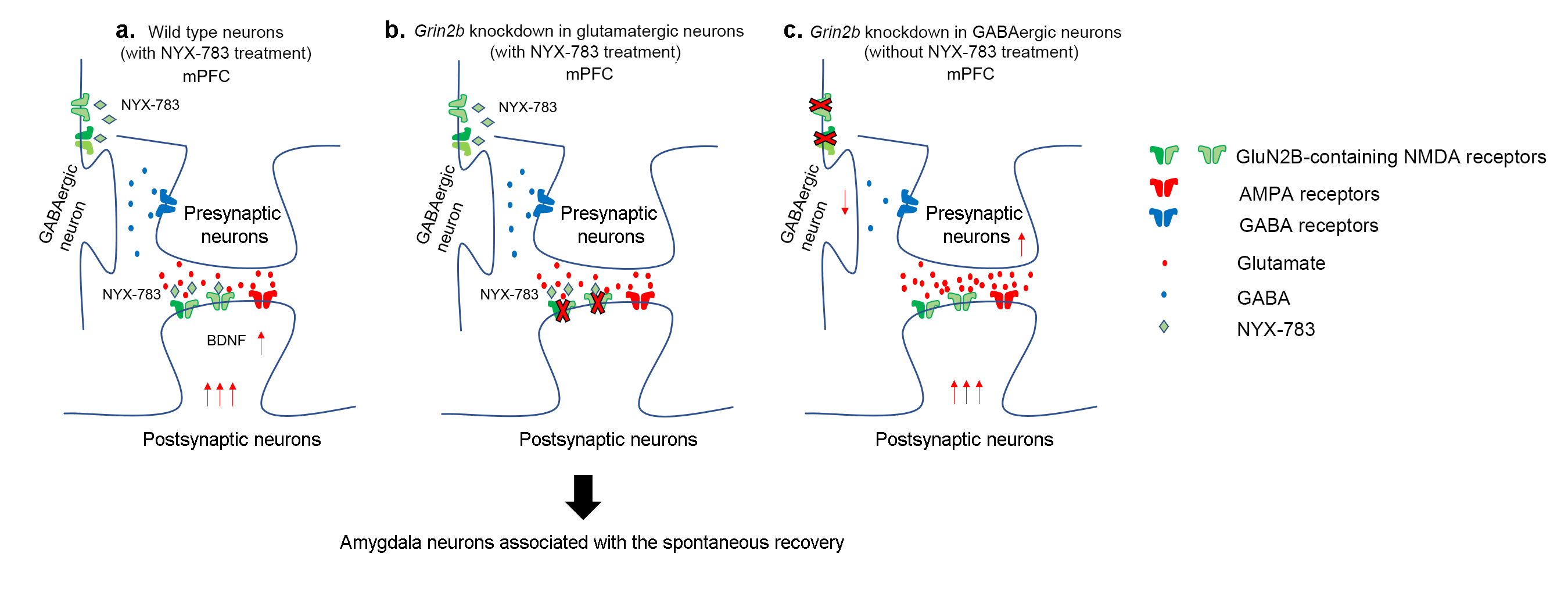

Supplement: Supplementary file 10 — Supplementary Figure 8. [file 41380_2022_1498_MOESM10_ESM.tif]
